# Prognostic evaluation of suicidality and development of risk factors in victims of physical and emotional child abuse and emotional/psychological neglect

**DOI:** 10.1192/bjo.2021.727

**Published:** 2021-06-18

**Authors:** Latif Miah

**Affiliations:** Swansea University Medical School, Morriston Hospital

## Abstract

**Aims:**

To evaluate the effects of childhood maltreatment, specifically physical and emotional abuse and emotional/psychological neglect on the development of suicidal ideation, depressive symptoms and self-harm. It is hypothesised that there will be a strong causal link between the aforementioned types of maltreatment with suicidality, depression and self-harm.

**Background:**

Child abuse is a major public health issue with profound developmental and mental health consequences towards victims and their contributions to society as a whole. The impact of sexual abuse is well-established, however non-sexual child maltreatment and its sequelae are not as well understood or studied.

**Method:**

A literature search was carried out using the Pubmed, Cochrane, Scopus and Google Scholar databases. Articles were appraised according to set criteria and manually screened for relevance to the review.

**Result:**

The results of this review demonstrate that there are statistically significant, potentially causal links between emotional and physical abuse, and emotional/psychological neglect with suicidal ideation, depressive symptoms and self-harm. More research is still required to elucidate the role of polyvictimisation in mental health outcomes and to further confirm these links between abuse and development.

**Conclusion:**

Child maltreatment remains a large public health issue with major impact on the economy of the world. It has profound, potentially lifelong consequences on victims and is something that needs to lose its stigma so that it can be identified earlier and potential damage prevented as far as possible. The future may lie in working to remove the stigma surrounding it, standardise how it is studied and thus learn to recognise the signs earlier – ideally leading to implementation of policy to get victims to safety, preventing unncecessary harm.

